# Evaluation of a nurse-led aftercare intervention for patients with head and neck cancer treated with radiotherapy and cisplatin or cetuximab

**DOI:** 10.1097/NCC.0000000000000983

**Published:** 2021-08-09

**Authors:** Cora Braat, Gerda M. Verduijn, Heleen A. van der Stege, Marinella P.J. Offerman, Mariëlle A.C. Peeters, AnneLoes van Staa, Wendy H. Oldenmenger

**Affiliations:** Department of Radiotherapy, Erasmus MC Cancer Institute, University Medical Center Rotterdam, the Netherlands; Department of Radiotherapy, Erasmus MC Cancer Institute, University Medical Center Rotterdam, the Netherlands; Research Center Innovations in Care, Rotterdam University of Applied Sciences, the Netherlands; Department of Head and Neck Oncology, Erasmus MC Cancer Institute, University Medical Center Rotterdam, the Netherlands; Research Center Innovations in Care, Rotterdam University of Applied Sciences, the Netherlands; Research Center Innovations in Care, Rotterdam University of Applied Sciences, the Netherlands; Department of Medical Oncology, Erasmus MC Cancer Institute, University Medical Center Rotterdam, the Netherlands

**Keywords:** Nurse-led Intervention, Self-management, Aftercare, Head-and-Neck cancer, Quality of Life, Quality of Care

## Abstract

**Background:**

The supportive needs for head and neck cancer (HNC) patients during the vulnerable period after treatment are not always met. Therefore, more professional support regarding physical, social and psychological care as well as lifestyle is recommended.

**Objective:**

Evaluation of a nurse-led aftercare intervention to support patients recovering from HNC treatment.

**Methods:**

Intervention group (IG) participants received two extra consultations from a nurse practitioner three and nine months after treatment for HNC. A holistic conversational tool, the Self-Management Web, was developed to guide the nurse through the conversation. Primary outcomes were health-related quality of life (HRQoL) and quality of patient-centered care. A secondary outcome was self-management skills.

**Results:**

27 patients were included in the IG and 28 in the control group (CG). Differences in HRQoL and self-management between the IG and CG were not statistically significant. For the IG, all domains of the Self-Management Web were perceived important and addressed by the nurse practitioner.

**Conclusion:**

This holistic nurse-led aftercare intervention was highly appreciated by HNC patients. Though the intervention met the need for support in recovery after treatment, it did not improve HRQoL or self-management skills.

**Implications of practice:**

For both nurses and patients the intervention is feasible and acceptable in daily practice. Self-management support for patients after their cancer treatment is of added value and has potential to improve the quality of regular follow-up care.

## Introduction

Worldwide head and neck carcinomas are the sixth leading cancer by incidence^1^. In the case of locally advanced head and neck carcinomas (HNC), organ-sparing treatment with the use of radiotherapy combined with cisplatin or cetuximab often is the preferred treatment^2, 3^. This type of treatment is associated, however, with high toxicity profiles^4^. The most common side-effect is disruption in the function and integrity of the mucosa of the mouth, which results in mucositis, manifesting itself anywhere from a mild burning sensation to large and painful ulcers that have a high impact on patients’ health related quality of life (HRQoL)^5^. Mucositis leads to a decrease in patients’ physical wellbeing, energy and strength^6^. The reduced swallowing function is a disturbing side-effect but it usually recovers during the first weeks and months after treatment. Other side-effects such as dry mouth, sticky saliva and functionality in opening one’s mouth can deteriorate significantly^7^. Apart from these physical impairments, high levels of psychological afflictions such as depression, anxiety, and distress are often observed^8, 9^. Additionally, HNC patients who are younger, have a lower socio-economic status, are unemployed, and those with self-reported comorbidity generally experience a poor HRQoL^10^. An earlier study of our research group showed that the acute and long-term side-effects can drastically influence patients’ daily lives^11^. HNC patients have to deal with both physical and psychosocial symptoms. Additionally, they have to build up their self-confidence, which is necessary to be able to resume their lives^11^. For many patients it is difficult to manage health-related chronic conditions and integrate them in their daily lives with the aim of achieving optimal QoL^12^.

People with cancer live longer and have to manage their cancer as a chronic illness^13–15^. Moreover, they are expected to be capable enough to take responsibility for their own care. Although self-management and self-regulation are useful frameworks, for oncology practices these concepts are still challenging models to empower cancer survivors. The concept of self-management has been defined into five core self-management skills: problem solving, decision making, resource utilization, forming a patient-health care provider partnership, and taking action^16^. Self-management support asks for an individualized approach per patient. To focus on patients’ intrinsic processes, a combination of four nursing perspectives is most effective, i.e. as coach, clinician, gatekeeper and educator^17^. Assessment of self-management abilities should play a role in the rehabilitation for HNC patients to help them in setting goals^9^.

During the vulnerable period after treatment, the supportive needs for HNC patients are not always being met^11^. More professional support is needed regarding physical care, lifestyle programs, social and psychological care^11, 18^. Such needs might be addressed by a practical, nurse-led self-management aftercare intervention during the initial post-treatment period^13, 18^. To develop such an intervention, we used the Intervention Mapping approach^20,21^. The key element of this intervention is coaching patients in developing problem-solving skills and self-confidence to enable them to take more control of their own rehabilitation^17, 19^. This study represents a first assessment of the effects of a nurse-led aftercare intervention for patients treated for head and neck cancer with radiotherapy combined with chemotherapy or cetuximab. Primary outcomes were HRQoL and quality of patient-centered care. Secondary outcomes were self-management skills.

## Methods

### Development of the nurse-led aftercare intervention

For the development of the intervention, we used the Intervention Mapping (IM) procedure^20, 21^ (https://interventionmapping.com). The IM protocol distinguishes six steps with corresponding tasks (Figure 1). In total, the development and implementation of the intervention took two years (2015-2017). The first four steps of the IM procedure were executed together with colleagues in kidney transplantation^19^ (Figure 1).

### Step 1: Needs Assessment

In this step, the needs of patients in treatment for HNC, nurses and nurse practitioners (NP) regarding self-management (support) were explored in several qualitative studies. First, we reviewed the qualitative literature on patients’ needs and preferences for self-management support^22^; this review revealed that for patients with chronic conditions, it is important that self-management support is tailored to their individual needs. Furthermore, they need information and instrumental, psychosocial, and relational support. Patients often reported that these needs were unmet because professionals focus on informational and instrumental support alone. Developing a collaborative partnership with shared decision making is key to improving self-management support^22^. This encouraged us to further assess the specific needs, preferences, and challenges with regard to self-management support of HNC patients in 2 focus groups and 6 individual interviews (total n=13)^11^. Most patients wished to receive professional support for dealing with post-treatment consequences. Apart from physical complaints, patients had difficulties in dealing with the emotional aspects of HNC and its treatment and struggled with building self-confidence to move on with their lives.

To explore nurses’ perceptions, attitudes, and potential needs, interviews were held and observations were performed. All participants worked at the same university medical hospital in various outpatient departments. Individual semi-structured interviews with nurses and NPs were held (n=27) to investigate nurses’ views on the concept of self-management in general and how these views related to the self-management interventions they use in clinical practice^23^. Results showed three distinct views on self-management support as follows: adhering to a medical regimen; monitoring symptoms; and integrating illness into daily life; only the last viewpoint reflected a holistic approach with the nurse focusing on coaching. Medical management was the focus of self-management for many nurses. The lack of attention for psychosocial aspects may be attributed to a lack of confidence, skills needed to address psychosocial issues, or available tools or interventions that limited them in offering psychosocial support. To more objectively assess NPs’ roles and skills in outpatient consultations and how this compared with their perception of their responsibilities for patients with chronic conditions, NPs (n=5) were observed during daily practice^24^. Although NPs reported that they considered building a relationship with their patients of utmost importance, their consultations were mostly based on a conventional medical model of medical history taking. Little attention was paid to the social, psychological, and behavioral dimensions of illness. Finally, a realist review of the literature was conducted to understand how nurse-led interventions that support self-management of patients with chronic conditions work and in what context they work successfully. Interventions focusing on intrinsic processes were found to be the most effective, as opposed to focusing solely on education^17^.

### Program Goals

Based on the needs assessment described above, we developed a nurse-led self-management support intervention that included the following key elements: a general, open structure that leaves room for individual preferences and tailoring of support; a holistic approach encompassing medical, emotional, and social self-management challenges; promoting shared decision making between nurses and patients; and patient empowerment by supporting self-efficacy and intrinsic motivation. The overall goal of the intervention is for patients with HNC to enhance their self-management skills to integrate their treatment and life goals and subsequently optimize their HRQoL and health-related outcomes. In addition, we aimed to improve NPs’ skills to optimize self-management support.

### Step 2: Matrices of Change Objectives

The second step of IM links the overall goals of the intervention to concrete actions by stating change objectives that specify who and what will change because of the intervention. To generate these change objectives, we combined performance objectives and the relevant determinants into a matrix. Change objectives were formulated both for patients and for NPs^19^. The performance objectives for NPs were guided by the Self-Regulation Theory^25^. In addition, the intervention focused on three components of the Five A’s model^26^, namely assessing behavior, beliefs, and motivation, agreeing with the patients on realistic goals, and assisting patients to anticipate barriers and develop a specific action plan.

### Step 3: Theory-Based Methods and Practical strategies

In Step 3, theory-based methods were selected and translated into practical strategies to influence each determinant in order to achieve the change objective. For example, techniques from Motivational Interviewing were used to promote motivation. Principles of Solution-Focused Brief Therapy^27^ were used for the goal and action-oriented change objectives.

### Step 4: Program Production

**Figure 2:**
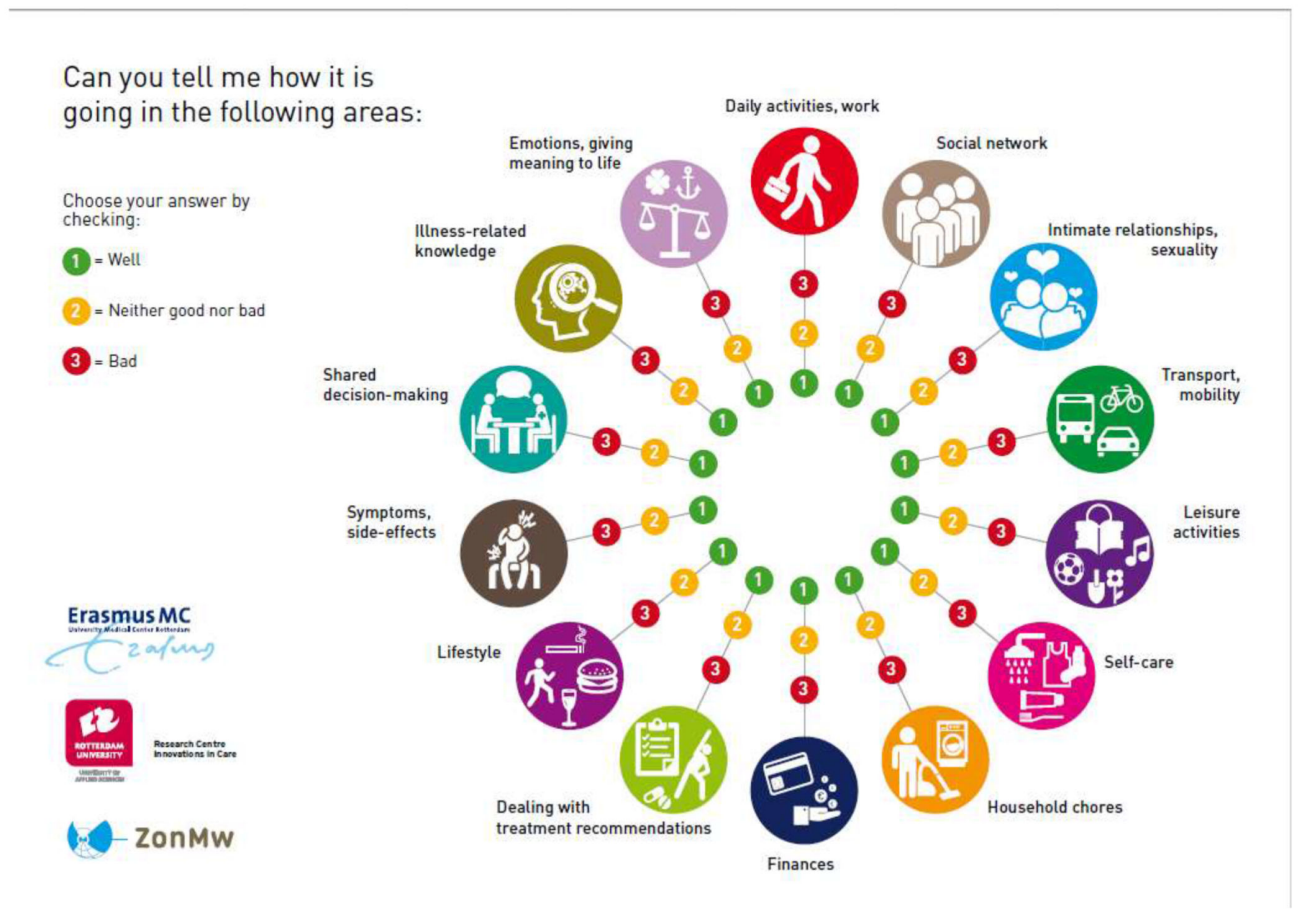
Self-Management Web

In Step 4, the actual program was developed. The intervention consisted of two extra, structured consultations with the NP with a duration of 30 minutes each. The conversations were based on solution-focused communication techniques, including goal-setting, action-planning and monitoring progress. To encourage the patient’s active participation, a visual communication tool was developed with patients and nurses. This ‘Self-management Web’^19^ (SMW) presents 14 icons related to every-day life domains (Figure 2). The goal of this tool was to encourage an open conversation between nurses and patients. Patients were in control to choose the area they preferred to focus on. By ranking the domains according to importance if they were doing well (1=green), neither good /nor bad (2=orange) or bad (3=red), they determined the content of the conversation. The patient encircled his answers on the SMW, and thus made the important issues for him to discuss explicit. Subsequently, in dialogue with the nurse the patient looked back on the treatment period and was encouraged to share the need for support to rebuild life after treatment. In case of a 2 or 3 answer on the web, the nurse asked open questions to clarify the problem. One week before the consultation, the SMW was sent to the patient’s home to be completed before consultation.

Recovering from challenging side-effects takes place in the first weeks to months after treatment. Therefore, the first nurse appointment was planned after the most demanding period. This initial conversation was focused on setting priorities, defining goals and making action plans for the future. After one month, the NP called the patient to evaluate these goals and to assess specific needs to reach the desired goals. During the second conversation, six months after the first one, the NP evaluated the goals and plans and assessed whether the patient had enough knowledge and skills to manage the illness and integrate these abilities in daily life. When necessary, the NP referred the patient to more specialized professionals.

### Step 5: Program implementation plan

All sections of the intervention were recorded in a protocol. The NP was trained by an experienced psychotherapist in skills of motivational interviewing, learned how to discuss the problems encountered and to apply techniques with which to convert these skills into practice. This concurred together with the NPs working in kidney transplantation. The NP also received feedback in order to improve the interviewing skills.

### Step 6: Evaluation plan

An evaluation protocol was developed consisting of a pilot study at the outpatient clinic of the Erasmus MC in Rotterdam, the Netherlands. Eligible patients for both the intervention group (IG) and the historical control group were diagnosed with HNC and were successfully treated with radiotherapy and chemotherapy or cetuximab, were older than 18 years of age, were able to read and speak Dutch, and had no current signs of illness. Three months after treatment, before the start of the intervention, recipients in the IG received a questionnaire by mail (T0) followed by a second questionnaire six months after completion of the intervention (T1). While not withholding patients from possible benefits of the patient navigation intervention we compared the IG with a retrospective historical control group HCG, who did not receive the intervention. The patients who ended their medical treatment between March and November 2015 were invited for the HCG in a consecutive order. When interested, they received an information letter, informed consent and the questionnaire.

Care as usual: All patients received the usual treatment and care, consisting of a weekly consultation during treatment at the outpatient clinic with a radiation oncologist alternating with NP to check and manage treatment-related side-effects. The purpose of the aftercare intervention was to improve patient-centered aftercare and empower patients in taking control of their rehabilitation from a holistic view. The intervention added two extra consultations with the NP to the usual care protocol, three and nine months after their treatment.

### Measurements

#### Quantitative data

HRQoL was measured with the validated Dutch version of the European Organization for Research and Treatment of Cancer Quality of Life Questionnaire 30 (EORTC QLQ-C30)^28^. The EORTC QLQ-C30 consists of five functional scales (physical, role, emotional, cognitive and social), a symptom scale and a global QoL scale. All the scales range in scores between 0-100. A high score on the functional scales represents a high degree of functioning, while a high score on the symptom scale represents a high level of symptom burden. On all scales the Cronbach’s alpha showed a coefficient of >0.70^28^. The additional EORTC QLQ Head and Neck 35 version is a disease-specific measure to assess HRQoL among patients with head and neck cancer. This 35-item questionnaire generates seven multiple-item scales in addition to eleven single item scales, with an adequate internal consistency^29^.

The Partners in Health (PIH) scale rates a patient’s self-management skills^30^. The Dutch PIH scale consists of two subscales: 1) ‘knowledge and coping’, and 2) ’recognition and management of symptoms and adherence to treatment’. The Cronbach’s alphas of the subscales were 0.80 and 0.72, respectively. The correlation between the subscales was 0.43^30^.

Self-efficacy was measured with the Self-Efficacy for Managing Chronic Disease 6-item scale (SECD-6), on a 10-point Likert scale, from 1 (not at all confident) to 10 (totally confident)^23^. The higher the score, the higher the degree of self-efficacy. The Cronbach’s alpha of the SECD-6 is 0.91^31^.

Patients’ experiences and appreciation in the quality of patient-centered care was measured with a subscale of the Consumer Assessment of Healthcare Plan Surveys (CAHPS)^24^. This subscale consists of five questions using a 5-point Likert scale from 1 (no, definitely not) to 4 (yes, definitely). The CAHPS is validated for the Dutch language (α=0.90)^32^.

Additionally, to evaluate delivered patient-centered care, we used a questionnaire based on the topics of the SMW. Patients indicated the importance of paying attention to various topics and the actual attention the NP paid to these topics^33^. The scale consists of 14 items scored on a 3-points Likert scale (importance items: 1=not important, 2=somewhat important, and 3=very important; attention items: 1=no attention, 2=some attention, 3=much attention). For the analysis, options 1 and 2 were recoded as negative and 3 as positive.

#### Qualitative data

To assess fidelity and feasibility of the intervention, an independent researcher interviewed six random patients of the IG who had completed the intervention. The interview questions focused on the topics: support from the NP during the aftercare consultations, the importance of discussing a broad range of issues related to medical, emotional and role management, and the use of the SMW.

In addition, to analyze fidelity six randomly chosen consultations were observed by an independent researcher. She was not involved in the study, works at the same department, and is an expert in qualitative research. Main topic of the observation was whether the NP adhered to the procedure of the intervention. Themes of the observations were: the introduction and use of the SMW, addressing needs for support, ‘where are you now’ and ‘where do you want to go’, confirming the motivation to change, focusing on positive outcomes and setting goals^19^.

### Data analysis

#### Qualitative analysis

Quantitative data were analyzed using IBM SPSS Statistics version 24.0. Patient demographics, clinical characteristics and questionnaires were summarized using descriptive statistics. Scores of all HRQoL-scales were transformed into a 0-100 scale. Scores for the QLQ-C30 and QLQ-H&N35 were calculated according to the EORTC scoring manual^28^. The outcome measurements are reported with medians and interquartile ranges (IQR). To analyze the impact of the intervention on patients’ HRQoL and self-efficacy, the Wilcoxon test was used for the baseline-follow-up analysis within the intervention group (T0-T1), and the Mann Whitney test was used for testing differences between the IG (T1) and HCG. Differences in the importance of the SMW topics within the IG were analyzed with the McNemar-Bowker test, and the difference between the IG and HCG in this respect was analyzed with the trend test. Effect sizes for the outcome measures were calculated with the bias-correct effect size Hedges (G). Effect size was interpreted as small (=0.20), medium (=0.50), or large (=0.80)^34^. A P-value ≤ .05 (2-sided) was considered statistically significant.

#### Qualitative analysis

All interviews were audio-recorded and were anonymized and transcribed verbatim. Qualitative data were summarized and discussed by two members of the research team. The observations were analyzed using a semi-structured observation protocol with topics related to elements of the intervention (use of the Self-Management Web by the NP, focus on positive results, and goal setting).

#### Ethical considerations

The study participants were informed about the study orally and in writing by the NP, and signed a document of informed consent. All participants were assured of confidentiality. Data were processed anonymously; only qualified researchers had access to the anonymized data. All data were stored on a password protected secured server within the firewall of the hospital. The study was approved by the medical ethics review board.

## Results

### Participant demographics

**Figure 3:**
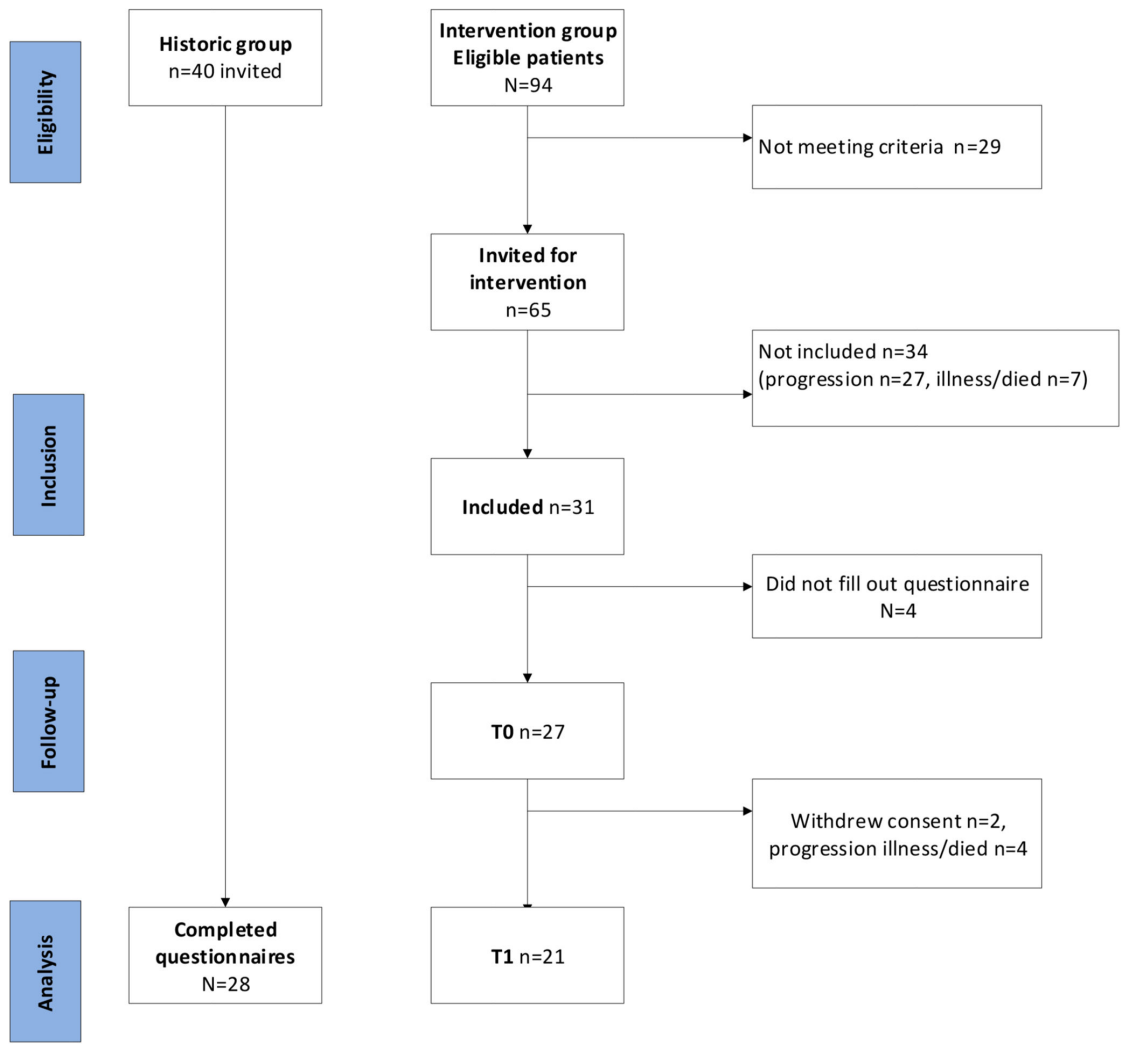
Flowchart inclusion

Before implementation of the intervention 40 patients were invited for the HCG, of whom 28 participated (enrollment rate 70%). Out of 94 eligible patients treated between November 2015 and November 2016, 65 were invited to participate in the IG. Of those, 38 (58%) were excluded because of disease progression (n=24), death before start of the intervention (n=7), or withdrawal of consent and/or non-response (n=7). Thus, 27 patients were included, of whom 6 dropped out during the study due to consent withdrawal (n=2) or death (n=4) (Figure 3). The median age of the patients included was 62 years in both groups; the majority were male (IG 85% vs. HCG 64%), and most patients had either oropharynx cancer (IG 48% vs. HCG 43%) or hypopharynx cancer (IG 41% vs. HCG 36%; Table 1).

#### Quality of life and quality of care

Patients’ scores for both cancer-related HRQoL and H&N specific QoL did not significantly differ between the IG and the HCG (Table 2). Within the IG, medium effect sizes on HRQoL were found on physical (.32) and cognitive (.27) functioning and symptoms scores (-.48).

Medium effect sizes were also found for pain (-0.51), trouble with social eating (-0.47), teeth (-0.64), dry mouth (-0.75), and sticky saliva (-0.65) from the H&N specific QoL. No differences were found in both groups in the level of confidence in patient-centered care measured with the CAHPS (Table 2). Analysis revealed no statistically significant differences in self-management skills and self-efficacy (Table 2).

#### Evaluation of the patient-centeredness of the aftercare intervention

**Figure 4:**
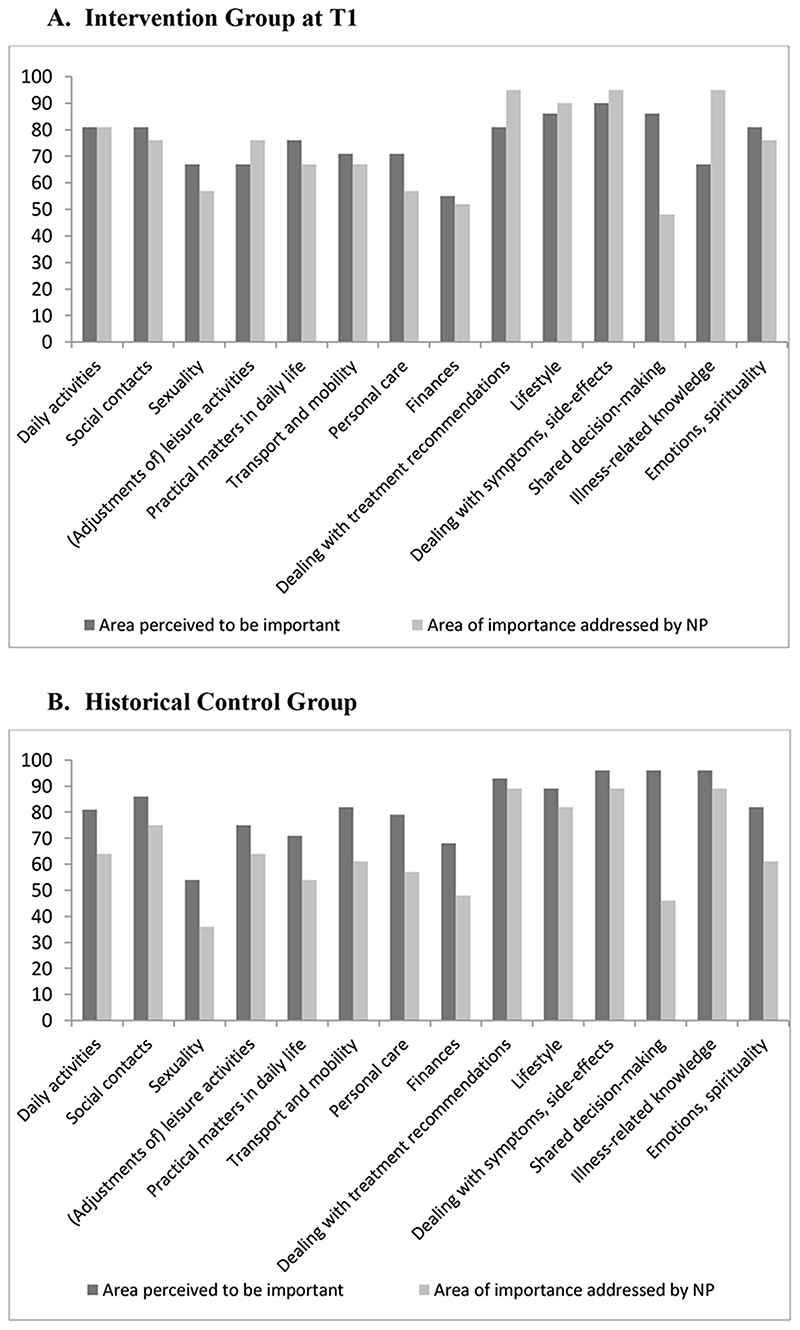
Importance of Paying Attention and Actual Paid Attention of the Domains of Care

Results of the questionnaire based on the topics of the SMW showed that for patients of the IG nearly all fourteen domains which rated as being important to discuss, were addressed by the NP. The top three most important domains were: dealing with treatment recommendations, lifestyle, and handling of symptoms and side-effects (Figure 4a). Remarkable finding was that for patients shared decision-making was perceived as being important, but only in half of the cases addressed by the NP. The opposite was the case for illness-related knowledge, which was less frequently perceived to be important to the patients, but was addressed more often. For the patients of the HCG, all domains of the SMW were perceived to be important to discuss (Figure 4b).

The six patients who were interviewed highly appreciated the aftercare intervention for discussing the fourteen domains of the SMW, in particularly the non-medical subjects. Patients indicated that the SMW form was comprehensive and they appreciated the broad range of topics of the SMW. *“For me it is a good thing, it gives you more overview and the Web makes it easier to discuss things”*. Another patient said, *“The NP is familiar with my total medical trajectory and she looks further than that”*. Patients deemed almost all themes to be important to discuss with the NP and stated that the SMW helps talking about difficult topics. One respondent explained: *“Filled out [the Web] twice. I answered the topic of intimate relationships differently the second time. The NP started a whole conversation about that; my wife and I benefited greatly from that”*. Concerning goal setting, one patient said, *“The NP emphasized that you should not immediately return to your old employment situation. We set goals about the reintegration”*. Some patients reported that during the period of aftercare, the support for their spouses could be improved. *“I did not need anything for myself, but my wife did”*. With respect to the frequency of the aftercare conversations, the answers ranged from *“two aftercare consultations are not enough”* to *“two is fine”*.

### Fidelity and feasibility

All patients in the intervention group received the interventions as intended. The observations of six consultations revealed that the NP delivered the intervention as intended. The NP discussed the results of the SMW and addressed needs and motivation to change. The NP encouraged the patients to choose feasible and concrete goals. Not all patients wished to discuss goals, because they were already working on their own personal goals, independent of the consultations: *“I do not need to discuss goals. I go for a walk and try to stay in shape”*.

## Discussion

This study represents the results of the preliminary assessment of the effects of a nurse-led aftercare intervention in supporting HNC patients to enhance their life after chemoradiation. The holistic approach in enabling patients to take more control of their rehabilitation discussing the fourteen domains of the SMW, was highly valued. The intervention was found to be feasible and acceptable by the nurse as well as with the patients. Within the IG, medium effect sizes were found in QoL although this could also be attributed to normal recovery during the first year after treatment. The intervention had no statistically significant effect on the patients between the IG and HCG regarding HRQoL nor self-management skills.

An aftercare intervention based on holistic principles implies a positive contribution to better personal care, which is important in supporting HNC patients’ self-management after their treatment. Recently, more nurse-led aftercare programs were evaluated in favor of self-management support^33, 35^. Additionally, the possibilities of web-based or online programs to enhance self-management skills have been explored in patients with HNC^18, 36, 37^. These online screening and monitoring tools were considered useful^37^, and patients with HNC expressed interest in using online programs^38^. However, computer-assisted supportive care and e-health programs are not feasible for all patients. For older and lower educated patients, personal counseling seems to be a more appropriate approach to address their specific needs^10^. Furthermore, considering the impact of long-term side-effects of HNC treatment like speech difficulties, tinnitus, and negative emotional consequences, individual support seems more appropriate for patients in practicing active self-management skills^8^. Another approved method to measure cancer patients’ well-being is the application of the Distress Thermometer and Problem List^39^. This tool is convenient to identify physical symptoms and stress related issues, but in comparing to our intervention less focused on developing patients’ self-management skills.

All patients in the study found the broad spectrum of daily life areas important to discuss. The patients in the HCG indicated that there was a lack of support for all domains, particularly regarding sexuality and shared decision-making. For patients in the IG, almost all the domains that were deemed important were addressed by the NP during the intervention. However, shared decision-making was less well implemented in daily practice. This topic is still an important theme to discuss, especially from the point of view of self-management strategies and considering patients have become more assertive in recent years. Attention was paid to the domain of illness-related knowledge. This agrees with Wilkie’s study, which concludes that treatment follow-up mostly focuses on physical aspects of recovery; however, this does not align with what is most important for the people concerned^40^. Domains such as daily activities, sexuality, emotions, and spirituality are discussed less often (Figure 4). The importance of emotional and social topics in supportive care after treatment has also been emphasized in other studies^11, 41^. Consequently, there is a risk that the needs of patients with HNC are overlooked or unresolved and therefore can lead to a lack of appropriate care and to fewer referrals to other professionals as well. This implies that the current aftercare protocol needs to be reorganized on a more comprehensive base^42^. Our study shows that the Self-Management Web can be used to implement a more holistic approach during the aftercare of the patients with HNC.

Another key element of the intervention was the advanced training in interview skills all involved NPs received. In the study of Been et al. among kidney patients, the NP’s reported that feedback about their skills helped them to improve their capability in conducting the interviews, what resulted in a significant improvement in delivered care between baseline and follow-up^33^. The fact that in our study no differences were found in patients’ experiences and appreciation in the quality of patient-centered care (CAHPS) might be caused by different reasons, e.g. the small sample size, the questionnaire itself, but might also be a consequence of the fact that the NP was already familiar with the patients during the treatment period. Therefore, it is possible there was no effort to continue the already existing relation although there was a gap of months between treatment and the study-related consultations. Nevertheless, problem-solving skills and self-confidence as well as empowering patients to take control of their rehabilitation is a meaningful aspect and should therefore be included in follow-up research.

## Limitations and strengths

A limitation of this pilot study is the small sample size, which precludes generalization of the findings. The low response rate may be explained by psychological reasons and the fact that people with a poor performance status are less willing to participate in clinical studies^43^. Furthermore, in the Netherlands, the care for patients with HNC is centered in specialized hospitals, and only patients of this one hospital were included. Another possible limitation is the use of a historic control group which might create a bias. However, during the years we conducted this study no changes have been made in the medical treatment nor in patients’ support, so both groups received similar care except our intervention. A strength of the design was the mixed-methods approach so that patients’ experiences with the intervention could be assessed. A strength of the intervention is that it was up to the patients to decide which domains were important for them personally. Lastly, the execution of this study was a realistic reflection of daily practice.

## Conclusion

This nurse-led intervention appears to be an appropriate intervention for nurses to support patients with HNC in their recovery process after treatment. In particular, the use of the SMW to reflect on rehabilitation was highly appreciated. In our opinion, self-management support for patients after their cancer treatment is of added value and has potential to improve the quality of regular follow-up care, even when significant improvements in HRQoL cannot be demonstrated.

## Figures and Tables

**Figure:**
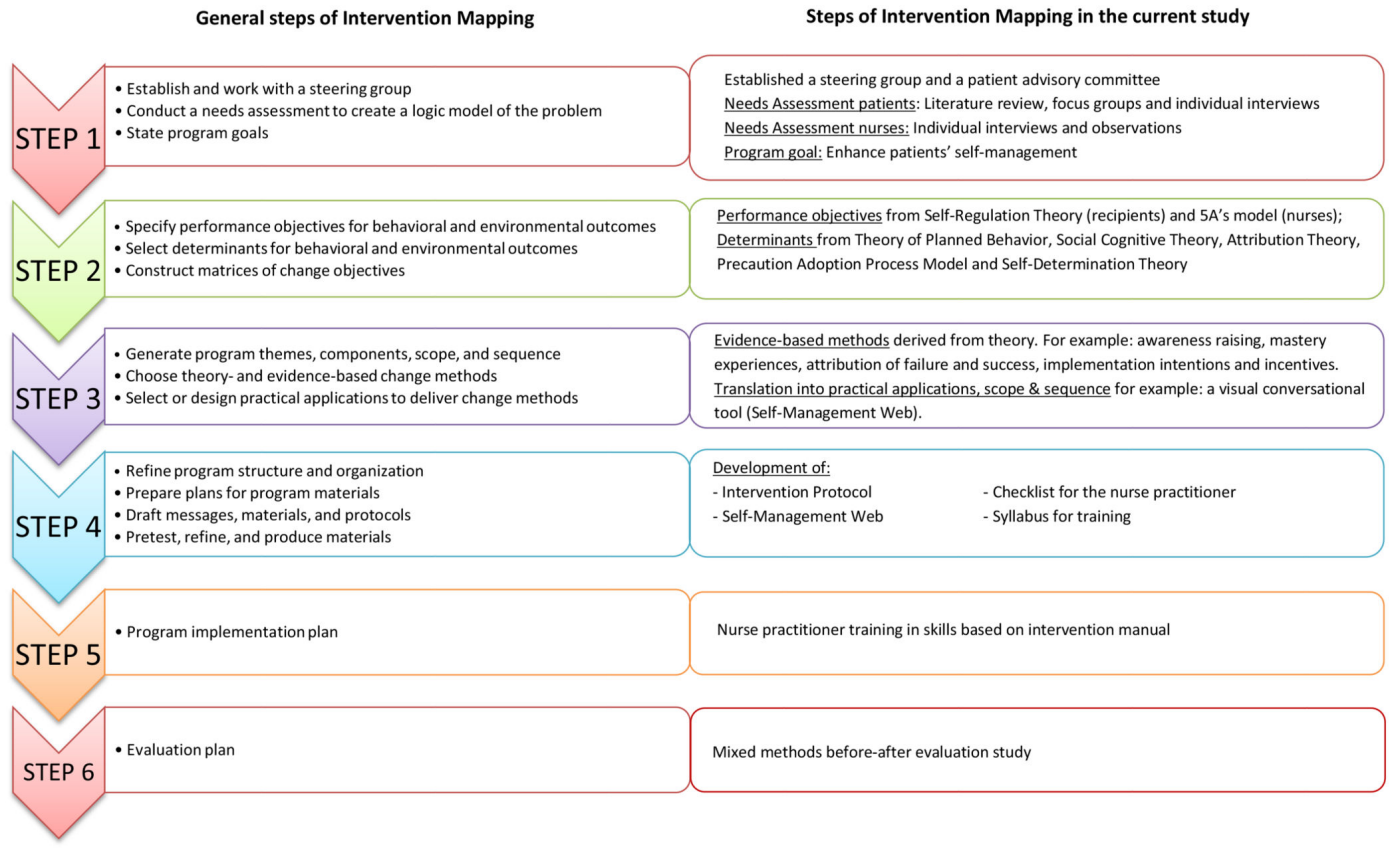


**Table 1:** Background Variables and Disease Characteristics

	Intervention Group n=27	Historical Control Group n=28
**Age, median, range**	62 (42-71)	62 (50-72)
**Gender, men *n (%)***	23 (85)	18 (64)
**Marital status *n (%)***		
**Single**	5 (19%)	9 (32%)
**Living together**	16 (59%)	19 (68%)
**Missing**	6 (22%)	0
**Cancer diagnosis *n (%)***		
**Oral cavity**	1 (4%)	4 (14%)
**Oropharynx**	13 (48%)	12 (43%)
**Hypopharynx**	11 (41%)	10 (36%)
**Nasopharynx**	1 (4%)	2 (7%)
**Other**	1 (4%)	0
**Treatment type *n (%)***		
**IMRT^1^ + CDDP^2^**	19 (90%)	23 (82%)
**IMRT + Cetuximab**	2 (10%)	5 (18%)

1 IMRT= Intensity-modulated radiotherapy

2 CDDP= Cisplatin

**Table 2:** Outcome Measurements

		Intervention Group	Intervention Group	Historical Control Group	p-value	Effect Size	
		T0, n=26	T1, n=21*	N=27	CG-IG	IG	CG-IG
		Median (IQR)	Median (IQR)	Median (IQR)		T0-T1	T1
**EORTC QLQ C30**								
**Quality of life**		79.2 (66.7-91.7)	83.3 (66.7-95.8)	83.3 (66.7-91.7)	.86	.11	.01
**Physical Functioning**		93.3 (80.0-100)	93.3 (86.7-100)	86.7 (73.3-100)	.17	.32	.41
**Role Functioning**		83.3 (66.7-100)	83.3 (50.0-100)	100 (70.8-100)	.19	-.04	-.52
**Emotional Functioning**		87.5 (66.7-100)	91.7 (62.5-91.7)	83.3 (66.7-100)	.99	.05	.14
**Cognitive Functioning**		100 (83.3-100)	100 (91.7-100)	83.3 (66.7-100)	**.023**	.27	.54
**Social Functioning**		83.3 (66.7-100)	100 (66.7-100)	83.3 (66.7-100)	.94	.12	.13
**Symptom Scores**		12.8 (5.1-24.4)	9.0 (1.9-15.4)	10.3 (5.1-23.1)	.53	-.48	-.31

**EORTC H&N 35**								
**Pain**		16.7 (8.3-41.7)	8.3 (8.3-22.9)	12.5 (0-27.1)	.97	-.51	-.18
**Swallowing**		8.3 (0-33.3)	4.2 (0-16.7)	16.7 (0-43.8)	.16	-.24	-.43
**Sense problems**		25.0 (12.5-33.3)	16.7 (8.3-33.3)	33.3 (0-50.0)	.42	-.24	-.34
**Speech problems**		22.2 (11.1-33.3)	11.1 (11.1-27.8)	11.1 (0-33.3)	.78	-.06	.12
**Trouble with social eating**		16.7 (8.3-41.7)	8.3 (0-25.0)^3^	29.2 (2.1-50.0)	.08	-.47	-.61
**Trouble with social contact**		0 (0-6.7)	0 (0-6.7)	0 (0-11.7)	.85	-.02	0
**Less sexuality**		16.7 (0-58.3)	0 (0-33.3)	0 (0-33.3)	.85	-.35	-.15
**Teeth**		0 (0-33.3)	0 (0-0)	0 (0-33.3)	.051	-.64	.60
**Opening mouth**		0 (0-33.3)	0 (0-33.3)	0 (0-66.7)	.47	.22	-.25
**Dry mouth**		66.7 (33.3-66.7)	33.3 (33.3-66.7)	66.7 (33.3-100)	.54	-.75	-.18
**Sticky saliva**		33.3 (33.3-75.0)	33.3 (0-50.0)^4^	33.3 (0-66.7)	.67	-.65	-.22
**Coughing**		33.3 (33.3-66.7)	33.3 (0-33.3)	33.3 (0-33.3)	.51	-.33	.12
**Felt ill**		0 (0-33.3)	0 (0-33.3)	0 (0-0)	.14	.05	.32
**Self-management knowledge and behavior (PIH)**								
**Knowledge and Coping**		6.6 (6.0-7.5)	7.0 (6.1-7.6)	6.9 (5.9-6.9)	.35	.29	.18
**Recognition and management of symptoms, adherence to treatment**		7.4 (5.8-7.7)	6.9 (5.4-8.0)	7.6 (6.4-8.0)	.58	-.28	-.38

**Self-efficacy**	**Total Score (SECD6)**	5.5 (3.4-7.3)	7.0 (4.8-9.2)	6.7 (4.0-8.0)	.26	-.15	.29


**Patient Centered care (CAHPS)**		4.0 (3.8-4.0)	4.0 (3.5-4.0)	4.0 (3.6-4.0)	.708	.62	.35

IQR=InterQuartileRange;

* EORTC QLQ C30: no significant differences within the IG;

1 IG T0-T1: p=.004;

2 IG T0-T1: p=.095;

3 IG T0-T1: p=.045;

4 IG T0-T1: p=.022;

4 CAHPS=Consumer Assessment of Health Plan Surveys. PIH=Partners in Health Scale; SECD6= Self-Efficacy for Managing Chronic Disease 6-item Scale.
